# Two sisters diagnosed with familial paraganglioma syndrome type 1 (FPGL1) and multiple endocrine neoplasia type 2A (MEN2A)

**DOI:** 10.1186/s12957-024-03418-1

**Published:** 2024-05-27

**Authors:** Katarzyna Stawarz, Monika Durzynska, Adam Galazka, Monika Paszkowska, Karolina Bienkowska-Pluta, Jakub Zwolinski, Andrzej Tysarowski, Ewa Kwiatkowska, Agnieszka Podgorska

**Affiliations:** 1 Head and Neck Cancer Department, Maria Sklodowska-Curie National Research Institute of Oncology, ul. W.K.Roentgen 5, Warsaw, 502-781 Poland; 2 Department of Pathology, Maria Sklodowska-Curie National Research Institute of Oncology, ul. W.K.Roentgen 5, Warsaw, 502-781 Poland; 3 Department of Genetics and Molecular Cancer Diagnostics, Maria Sklodowska-Curie National Research Institute of Oncology, ul. W.K.Roentgen 5, Warsaw, 502-781 Poland

## Abstract

**Background:**

In clinical practice, genetic testing has become standard for many cancerous diseases. While a diagnosis of a single hereditary syndrome is not uncommon, the coexistence of two genetic diseases, even with partially common symptoms, remains unusual. Therefore, targeted next-generation sequencing (NGS), along with genetic consultation and imaging studies, is essential for every patient with confirmed paraganglioma. In this report, we present two sisters diagnosed with multiple endocrine neoplasia type 2 (MEN2A) and familial paraganglioma syndrome type 1 (FPGL1).

**Case presentation:**

After presenting to the clinic with neck tumors persisting for several months, both patients underwent tumor removal procedures following imaging and laboratory studies. Pathological reports confirmed the diagnosis of paragangliomas. Subsequently, genetic testing, including NGS, revealed a mutation in the rearranged during transfection (*RET*) gene: the heterozygous change (c.2410G > A), (p.Val804Met), and a variant of the succinate dehydrogenase complex subunit D (*SDHD*) gene: (c.64 C > T), (p.Arg22Ter). Subsequently, thyroidectomy procedures were scheduled in both cases.

**Conclusion:**

To the best of our knowledge, this is the first report presenting these two mutations in two related patients, resulting in distinctive genetic syndromes with similar manifestations. This underscores that although infrequent, multiple hereditary disorders may co-occur in the same individual.

## Introduction

In clinical practice, genetic testing is now the standard of care for numerous cancerous conditions. While a diagnosis of a single hereditary syndrome is not uncommon, the coexistence of multiple genetic diseases, even with partially common symptoms, remains unusual. The occurrence of an individual with two separate heritable conditions accounts for approximately 4.6 to 7% [1]. In these case scenarios, we have described two sisters diagnosed with familial paraganglioma syndrome type 1 (FPGL1) and multiple endocrine neoplasia type 2 (MEN2A). MEN2A represents 70–80% of all MEN2 cases, with an estimated incidence of 1 in 30,000 [2, 3]. It’s inherited in an autosomal dominant manner and features medullary thyroid carcinoma, hyperparathyroidism, and pheochromocytoma [4, 5]. The condition arises from a *RET* (rearranged during transfection variant) [6] gene variant on chromosome 10q11.2, which often results in medullary thyroid carcinoma, typically diagnosed before 35 years [7–9]. Pheochromocytoma, another manifestation of MEN2A, affects fewer than half of patients, yet bilateral pheochromocytoma in MEN2A is not uncommon, occurring in 50–80% of cases [10, 11]. It typically remains asymptomatic or manifests years after the diagnosis of thyroid cancer [12, 13]. Another condition associated with the occurrence of pheochromocytoma is FPGL1, resulting from *SDHD* (succinate dehydrogenase complex subunit D) [14] gene mutations. This syndrome is characterized by non-secretory tumors located along the paravertebral axis, commonly found in the head and neck region [15–17], with an estimated incidence of 1–9 cases per 1,000,000 [18, 19]. While both MEN2A and FPGL1 syndrome are rare separate conditions, they may have overlapping features with different timing manifestations. However, RET gene mutations do not contribute to the tumorigenesis of neck paragangliomas. Nevertheless, thorough gene sequencing with genetic consultation, along with imaging studies, seems to play a crucial role in the evaluation of every patient with a confirmed paraganglioma. Herein, we present an extremely rare case report series of two sisters diagnosed with MEN2A and FPGL1 syndrome.

### Patient 1

**Fig. 1:**
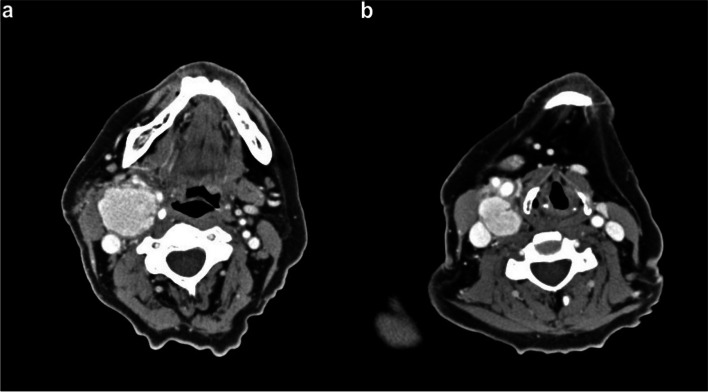
CT scan of a tumor in patient 1.** a **and** b** Horizontal CT scans of a well-defined tumor with robust, homogeneous contrast enhancement, measuring 34 × 31 mm in the transverse plane and approximately 60 mm in the craniocaudal dimension on the right side of the neck

**Fig. 2:**
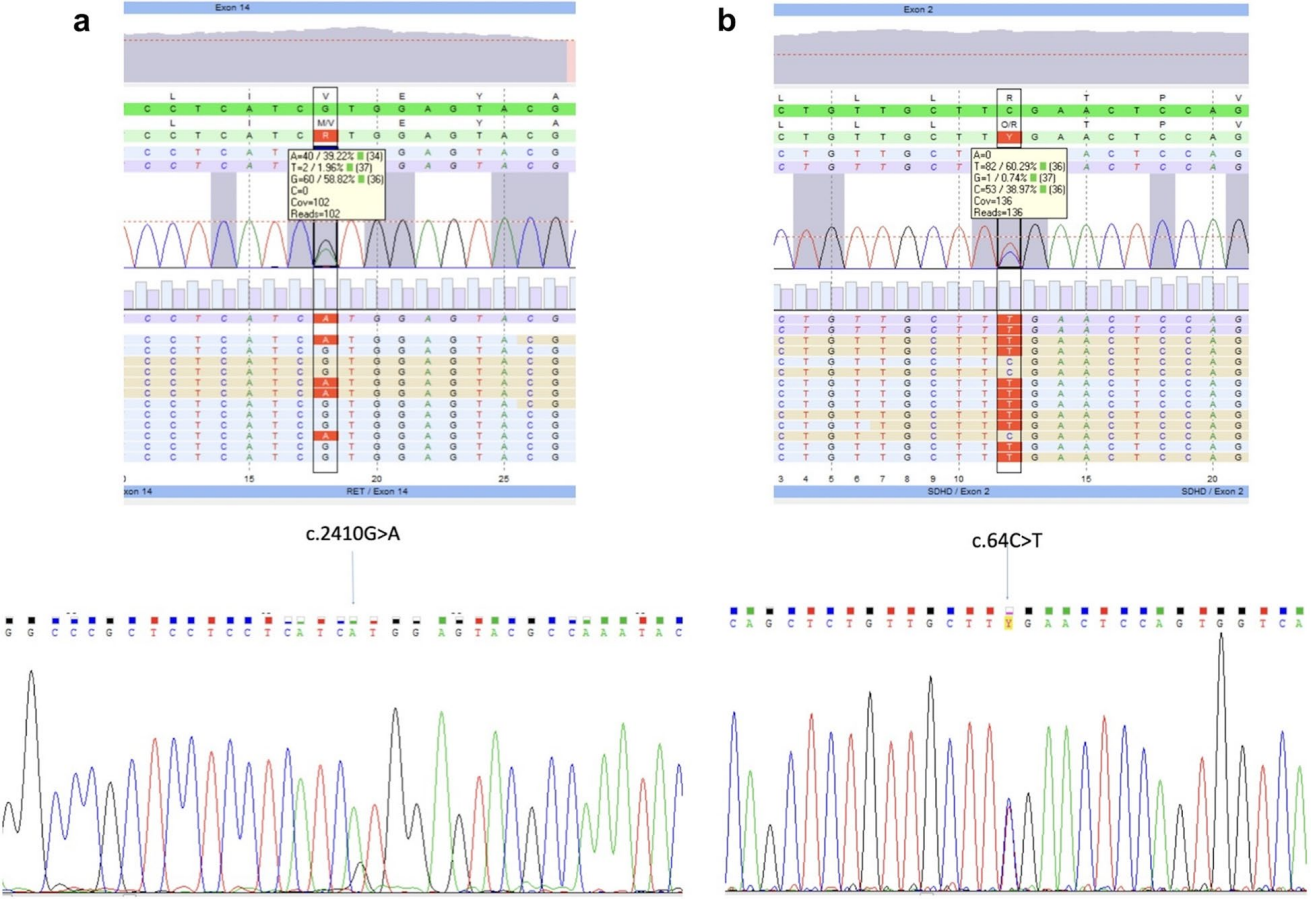
Results of next generation sequencing in two patients. SeqPilot program view of NGS sequencing and chromatogram from Sanger direct sequencing of the (**a**) RET and (**b**) SDHD gene

A 30-year-old woman of Polish descent presented to the Head and Neck Cancer Clinic of the National Research Institute of Oncology in Warsaw with a three-month history of a mass on the right side of her neck. She also complained of dizziness, hearing problems, difficulty swallowing, and abdominal pain, which had been worsening in frequency and severity. She had normal bowel movements and denied smoking or drinking alcohol. Her medical history was unremarkable, except for the loss of her parents in a car accident when she was 5 years old. There was no family history of genetic, metabolic, or neoplastic disorders. Concerned about the neck tumor and abdominal pain, she mentioned plans to conceive and attributed the symptoms to a possible infectious condition due to her job as a teacher. The basic laboratory tests, including WBC (white blood cells), RBC (red blood cells), HGB (hemoglobin), protein, and calcium levels, were within normal limits. Her vital signs were also normal. The physical examination revealed a right-sided neck mass of 6 centimeters in diameter. The mass was pulsatile, but the overlying skin was normal in color, texture, and temperature. Abdominal examination did not reveal any abnormality, as the abdomen was soft on palpation with a good peristaltic wave. The patient was then scheduled for a neck ultrasound and a CT scan of her body, including the head, neck, thorax, abdominal cavity, and pelvis. The ultrasound exam revealed a rounded mass with mixed echogenicity and well-defined margins, localized in the carotid bifurcation on the right side. The color Doppler function displayed hypervascularity of the tumor, so the biopsy was postponed. On the CT scans at the level of the bifurcation of the right common carotid artery and behind it, a fairly well-defined tumor was noticed, with strong, homogeneous contrast enhancement, measuring 34 × 31 mm in the transverse plane and approximately 60 mm in the cc dimension (Fig. 1). The tumor modeled the vascular structures but did not infiltrate them. No other abnormalities were detected on imaging studies, and the basic blood work-up proved to be normal. Subsequently, the patient underwent neck surgery, during which the tumor was removed with prior vascular embolization, with an uncomplicated post-surgical recovery. The histopathology results confirmed the diagnosis of a paraganglioma tumor with rounded cells with eosinophilic cytoplasm, and regular centralized nuclei. The proliferative index, evaluated by the Ki67 expression, was 1–3%. Expression of chromogranin, synaptophysin, and S100 protein were detected, but no somatic mutations in the tumor tissue were found. Due to the patient’s young age and the unknown family history of hereditary disorders, genetic testing was performed to rule out the hereditary origin of the tumor. Targeted next-generation sequencing was conducted following this protocol. DNA extraction was performed on 200 µl of whole blood (anti-coagulated with EDTA) and the genomic DNA isolation was carried out using the QIA symphony instrument from QIAGEN GmbH. The gene panel included, among others, *RET, MEN1, SDHA, SDHAF2, SDHC, SDHD* genes and was then employed to identify pathogenic changes in all coding exons and 30 bp of flanking non-coding sequences, using Roche’s SeqCap EZ HyperCap kit. Subsequently, sequencing was performed using an Illumina MiniSeq device. The results underwent analysis through the bioinformatics tools of the JSI SeqPilot platform, ensuring a minimum average coverage of gene coding sequences not less than 100 reads. The analysis included the identification of single nucleotide changes and multi-nucleotide deletions/insertions in gene coding sequences. The notation of changes/mutations followed the Human Genome Variation Society (HGVS) nomenclature. The presence of the identified pathogenic variants was confirmed by Sanger direct sequencing on the 3500 Genetic Analyzer Applied Biosystems/HITACHI. The NGS screening revealed a pathogenic *SDHD* variant - c.64 C > T (p.Arg22Ter) and a pathogenic *RET* variant: c.2410G > A (p.Val804Met). The RET gene heterozygous change c.2410G > A (p. Val804Met) is documented in the ClinVar (ID37102) and HGMD (CM981707) databases. Additionally, a SDHD gene heterozygous change c.64 C > T (p.Arg22Ter) is also documented in the ClinVar (ID6903) and HGMD (CM13282) databases. The SeqPilot program view of NGS sequencing and the chromatogram from Sanger direct sequencing of the detected variants, is presented in Fig. 2a and b. Based on the test results, the tumor appeared to have a genetic origin, necessitating a multidisciplinary approach. Therefore, the patient was referred for a genetic consult. Next-generation sequencing confirmed only two variants in *SDHD* and *RET* genes, respectively, with no abnormalities in *MAX, VHL*, or other *SDH* genes. Further diagnostic workup was ordered by a genetics specialist to confirm the diagnosis of FPGL1 syndrome and MEN2A as well. Regarding the *RET* variant, the patient subsequently underwent an ultrasonographic evaluation of the thyroid gland, which did not detect any thyroid or parathyroid abnormalities at that time. Nevertheless, the endocrinology consultation recommended thyroidectomy due to the significant risk of medullary thyroid carcinoma. Complete blood tests were ordered, including assessments of calcium levels, calcitonin, parathormone, thyroid stimulating hormone (TSH), carcinoembryonic antigen (CEA), complete blood count, vitamin D levels, estradiol, adrenocorticotropin, dehydroepiandrosterone sulfate, chromogranin A, testosterone, insulin, glucose, androstenedione, and cortisol. Only the calcitonin level showed a slight elevation, reaching about 15.8 pg/ml (Table 1). Daily urine collection, including urine catecholamines, was also conducted, revealing normal results (Table 1). Although imaging studies did not reveal any abnormalities besides the neck paraganglioma, the patient’s history of periodic abdominal pain prompted the necessity of imaging with [131I]/[123I] metaiodobenzylguanidine (MIBG), which ultimately yielded results within normal limits. With pheochromocytoma ruled out, the patient underwent a thyroidectomy procedure, which was successful and the recovery was uneventful. Due to the increased risk of gastrointestinal stromal tumor (GIST) associated with the inherited *SDHD* variant, the patient also underwent a gastroscopic exam in conjunction with an ultrasonographic exam four weeks after the thyroidectomy. The endoscopy revealed no findings suggestive of GIST. Nevertheless, the genetic variants of *RET* and *SDHD* genes place the patient at a lifetime risk of various cancers. Therefore, detailed follow-up tests are of utmost importance. An annual blood biochemical work-up, including plasma metanephrines, calcitonin, parathormone, and calcium levels, was ordered. Moreover, a 24-hour urine collection was also recommended to search for elevated fractionated metanephrines and catecholamines. Imaging studies, such as a total body CT scan with computed tomography angiography (CTA) examination, were performed once every two years. The patient has remained under close supervision of the National Institute of Oncology and has not developed any signs of hyperparathyroidism, pheochromocytoma, GIST, or other malignancies. Follow-up visits were scheduled every 3 and 6 months during the first and second year after diagnosis, respectively. Furthermore, genetic tests were offered for the patient’s first-line relatives. Although the patient seemed stressed about the inherited genetic conditions, she remained grateful as the diagnosis was made early, and the patient proceeded with the appropriate treatment plan.

**Table 1:** Results from laboratory tests of both presented patients

	Patient 1	Patient 2	Normal Value
**Gender**	Female	Female	-
**Calcitonin**	15.8	0.82	< 6.40 [pg/mL]
**Parathormone**	24.12	33.86	15–65 [pg/mL]
**Adrenocorticotropin**	8.1	9.3	7.2–63.6 [pg/mL]
**TSH**	1.390	1.060	0.300–4.200 [ulU/mL]
**FT3**	2.58	3.34	2.00-4.40 [pg/mL]
**FT4**	1.36	1.22	0.93–1.70 [ng/dL]
**Estradiol**	10.4	9.3	7.2–63.6 [pg/mL]
**Dehydroepiandrosterone sulfate**	177.70	169.60	60.90–337.00 [ug/dL]
**Chromogranin A**	48.3	57.09	< 101.9 [ng/mL]
**Testosterone**	10.5	13.4	8.4–48.1 [ng/dL]
**Insulin**	7.2	8.50	2.60–24.90 [ulU/mL]
**Androstendione**	0.878	0.999	0.490–1.310 [ng/mL]
**Cortisol**	2.01	2.04	Morning: 4.82–19.5 2.47–11.9 [ug/dL]
**CEA**	0.7	2.16	< 5.0 [ng/mL]
**Calcium**	2.52	2.33	2.20–2.65 [ mmol/L]
**Phosphorus**	1.09	1.21	0.81–1.45[ mmol/L]
**Vitamin D**	13.55	62.89	30–50 [ng/mL]
**Glucose**	81	98	70–99 [mg/dL]
**Plasma Dopamine**	2.3	3.1	0–30 [pg/mL]
**Plasma Epinephrine**	12.4	15.3	0–140 [pg/mL]
**Plasma Norepinephrine**	145.8	136.2	70–1700 [pg/mL]
**Plasma Metanephrine**	334	421.2	< 1180 [pmol/L]
**Plasma Normetanephrine**	233.1	178	< 510 [pmol/L]
**Plasma 3-methoxytyramine**	56.3	67.2	< 180 [pmol/L]
**Urine Dopamine**	490	511	420–2612 [nmol/24 hours]
**Urine Metanephrine**	245.5	221	140–785 [mcg/24 hours]
**Urine Epinephrine**	5.4	6.1	0.5–20 [mcg/24 hours]

### Patient 2

**Fig. 3:**
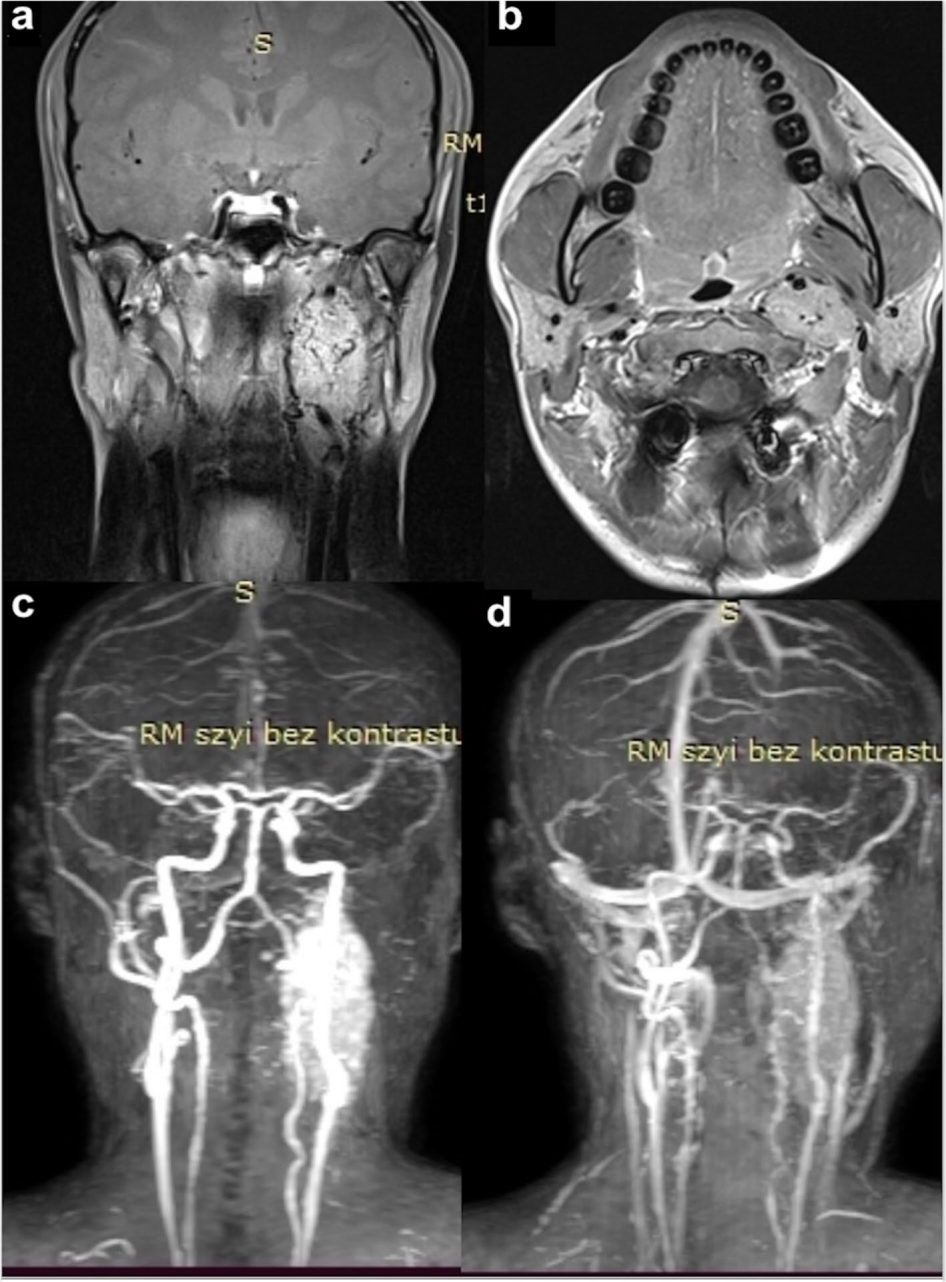
MRI and CTA imaging of a left-sided neck tumor in the patient 2. **a** coronal MRI, **b** horizontal MRI and **c**, **d** CTA scans displaying the round hypervascular tumor in the left common carotid artery bifurcation of 56 mm in the largest dimension

A 39-year-old woman of Polish origin, who was a sister of Patient 1, was seen in the Head and Neck Cancer Clinic based on the recommendation for genetic screening tests. The patient did not present any symptoms and had no significant past medical history. Similarly, the parents of this patient died in a car crash during her childhood, and there was no information about genetic or neoplastic disorders in the family. She did not smoke cigarettes or consume alcohol and led a healthy lifestyle as a dietitian. During the physical examination, a palpable mass on the left side of her neck was detected, but the examination was otherwise negative for any other abnormalities. The skin overlying the mass appeared unchanged, and her vital signs were within normal limits. Based on the detection of *RET* and *SDHD* gene variants in the patient’s sister, genetic screening with targeted NGS, following the same protocol, was also performed. The results revealed changes in the same genes as her sister’s: pathogenic *SDHD* variant - c.64 C > T (p.Arg22Ter) and pathogenic *RET* variant: c.2410G > A (p.Val804Met). Based on these findings, a diagnosis of MEN2A and FPGL1 syndrome was made. Laboratory tests, including calcium, calcitonin, parathormone, TSH, CEA, blood count, vitamin D level, estradiol, adrenocorticotropin, dehydroepiandrosterone sulfate, chromogranin A, testosterone, insulin, glucose, androstenedione, and cortisol, were ordered, in addition to a basic complete blood count (Table 1). Urine collection was also ordered but turned out to be within normal limits (Table 1). The patient underwent a neck ultrasound, computed tomography angiography (CTA) of the head and neck region, and a whole body MRI scan. Both CTA and an MRI scan revealed a round hyper-vascular tumor in the left common carotid artery bifurcation measuring 56 mm in the largest dimension (Fig. 3). The tumor was removed during the open biopsy procedure, which was preceded by a vascular embolization. The histopathological report confirmed the diagnosis of paraganglioma, with no somatic mutations detected in the tumor tissue. Subsequently, owing to the RET gene variant, the patient was scheduled for a thyroid ultrasound, which did not reveal any abnormality in both the thyroid or parathyroid glands. After an endocrinological consultation, a future prophylactic thyroidectomy might be recommended if elevated calcitonin levels or thyroid abnormalities are detected on ultrasound. The diagnostic and treatment approach provided were similar to the patient’s sister’s medical management. The patient remained under close observation at the National Institute of Oncology, as the genetic mutations in these two genes put her at a significant risk of developing malignancy. One month after the surgical paraganglioma removal, the patient was scheduled for regular follow-up visits, including gastroscopy to rule out GIST. Moreover, the CT/CTA scans were ordered every two years, with no findings of new tumors detected so far. She was seen in the clinic every 3 months during the first year of diagnosis and every 6 months in the second year. Although slightly confused and overwhelmed due to this serious diagnosis, the patient seemed to feel well taken care of.

## Discussion

The presented case reports offer unique examples of the rare co-occurrence of two genetic syndromes in two first-degree relatives. The available literature provides limited data on the simultaneous presence of multiple hereditary conditions in a single patient, particularly among relatives [20]. While rare, the co-occurrence of multiple genetic disorders in a single individual is possible. The case reports of two sisters demonstrate that shared symptoms may arise from distinct hereditary conditions with overlapping features. The presence of multiple paraganglioma tumors, particularly in extra-adrenal sites, should prompt suspicion of their genetic etiology. Typically, these tumors are benign, parasympathetic, and non-secretory, lacking metastatic potential. Symptoms primarily arise from their mass effect, manifesting as tinnitus, dizziness, or hearing impairment [21, 22]. However, around 10% of paraganglioma tumors, especially those located in extra-adrenal sites such as the bifurcation of the common carotid artery (CCA) in the head and neck region, demonstrate malignancy [23, 24]. In the presented cases, the paraganglioma tumors manifested as part of FPGL1 syndrome. FPGL1 arises from a mutation in the *SDHD* gene, which encodes the succinate dehydrogenase subunit. The succinate dehydrogenase enzyme, integral to mitochondrial complex II, plays a pivotal role in the Krebs cycle by catalyzing the conversion of succinate to fumarate, a critical step in electron transfer. However, mutations in the *SDHD* gene disrupt this process, leading to the accumulation of succinate within cells. Consequently, this accumulation triggers over-expression of hypoxia-induced factor-1 (HIF-1), which drives tumorigenesis, oxidative stress, and genomic instability [25]. The mutation commonly occurs in one allele, leading to loss of heterozygosity in the other wild-type allele, resulting in complete loss of enzyme function. Additionally, *SDHD* is maternally imprinted, meaning the disease predominantly arises when there is a mutation in the father’s gene, although generation skipping may also occur [26]. In the described cases, the only symptom associated with the *SDHD* mutation was the presence of a neck paraganglioma tumor, with no evidence of pheochromocytoma. However, another hereditary syndrome known to be associated with pheochromocytoma tumors is MEN2A [27], although the manifestation of its symptoms in the presented cases was limited. The presented patients were also identified with a *RET* gene variant linked to a significant risk of medullary thyroid carcinoma [28]. Nevertheless, the *RET* mutation discovered in the presented cases appears to be an incidental finding revealed by panel NGS analysis, as no obvious symptoms were reported by either patient. Furthermore, considering the firmly established genotype-phenotype correlation in MEN2A, the mild or absent manifestation of MEN2A in these two siblings may be linked to the specific *RET* gene variant identified. Elevated calcitonin levels detected in the first patient may precede macroscopic changes in the thyroid gland, indicating tumorigenesis [29]. While medullary thyroid cancer is the leading cause of mortality in MEN2A, determining the timing of prophylactic thyroidectomy is challenging due to varying symptoms among patients [30]. Pheochromocytoma, another manifestation of MEN2A, is typically benign [31] and its diagnosis relies mainly on imaging and laboratory assessments, including plasma and urinary catecholamine concentrations [32, 33]. While pheochromocytoma is not the predominant manifestation of either MEN2A or FPGL1 syndrome and was not identified in the presented patients, it serves as the common denominator linking these two disorders. Similarly, parathyroid abnormalities, although the least frequent manifestation of MEN2A [34] were also not observed in the presented cases. The presented case scenarios underscore the importance of thoroughly assessing complex or atypical presentations of rare symptoms to identify possible genetic multi-morbidities. To the best of our knowledge, the coincidence of these two variants in the *RET* and *SDHD* genes has not been previously reported. According to the Genome Aggregation Consortium (gnomAD), the pathogenic *RET* variant described here has been identified in 196/16,095,504 chromosomes in the general population [35]. Conversely, the *SDHD* gene variant described in the presented cases, was identified in 1/1,459,576 chromosomes in the global population [36]. Additionally, the RET gene heterozygous change (c.2410G > A), (p.Val804Met) stands as the most prevalent pathogenic variant associated with medullary thyroid carcinoma [37]. A recent investigation using the Examination Aggregation Consortium (ExAC) dataset estimated the penetrance of medullary thyroid carcinoma to be approximately 4% among *RET* variant (c.2410G > A), (p.Val804Met) carriers [38]. In contrast, another study involving 160 subjects with *RET* change (c.2410G > A), (p.Val804Met) indicated the penetrance of medullary thyroid carcinoma to be approximately 85% by the age of 70 [39]. On the other hand, due to the limited data on this particular SDHD gene variant provided in the gnomAD database, it seems challenging to assess the penetrance of this mutation. However, a comprehensive study conducted by Neumann et al. found that germline mutations of SDHD conferred a 50% penetrance by the age of 31 and 86% by the age of 50 [18].

Therefore, these data confirm that these two syndromes remain rare separate conditions, with their concomitant incidence not previously reported in the available literature. Nevertheless, despite the exceptional presentation of the described cases, the study is not without some limitations. The most significant limitation is the lack of genetic analysis conducted in other family members, including siblings, parents, grandparents, and other relatives. While it is highly recommended to perform genetic analyses in all family members, this approach may prove challenging due to limited availability of genetic screening and infrastructure in some hospitals.

## Conclusion

These case reports document, for the first time, the concurrent occurrence of *RET* gene variant (c.2410G > A), (p.Val804Met), and *SDHD* gene variant (c.64 C > T), (p.Arg22Ter) in two related individuals. Thus, these findings underscore that although rare, the simultaneous manifestation of multiple hereditary disorders within a single patient is indeed plausible.

## Data Availability

No datasets were generated or analysed during the current study.

## Abbreviations

CCA: Common carotid artery
CEA: Carcinoembryonic antigen
CTA: Computed tomography angiography
FPGL1: Familial paraganglioma syndrome type 1
GIST: Gastrointestinal stromal tumor
HGB: Hemoglobin
HGVS: Human Genome Variation Society
HIF-1: Hypoxia-induced factor-1
MEN2A: Multiple endocrine neoplasia type 2A
MIBG: Metaiodobenzylguanidine
NGS: Next generation sequencing
RBC: Red blood cells
RET: Rearranged during transfection
SDHD: Succinate dehydrogenase complex subunit D
TSH: Thyroid-stimulating hormone
WBC: White blood cells

## References

[1] Ferrer A, Schultz-Rogers L, Kaiwar C (2019). Three rare disease diagnoses in one patient through exome sequencing. Cold Spring Harb Mol Case Stud.

[2] Zbuk KM, Eng C (2007). Cancer phenomics: RET and PTEN as illustrative models. Nat Rev Cancer.

[3] Marini F, Falchetti A, Del Monte F (2006). Multiple endocrine neoplasia type 2. Orphanet J Rare Dis.

[4] Jessica Moline C, Eng. Multiple endocrine neoplasia type 2: an overview, Genet Med. 2011;13(9):755–64. 10.1097/GIM.0b013e318216cc6d. 10.1097/GIM.0b013e318216cc6d 21552134

[5] Subbiah V (2020). State-of-the-art strategies for targeting RET-dependent cancers.J. Clin Oncol.

[6] Bhattarai C, Poudel PP, Ghosh A, Kalthur SG (2022). The *RET* gene encodes RET protein, which triggers intracellular signaling pathways for enteric neurogenesis, and *RET* mutation results in Hirschsprung’s disease. AIMS Neurosci.

[7] Sonali, Thosani et al. The Characterization of Pheochromocytoma and Its Impact on Overall Survival in Multiple Endocrine Neoplasia Type 2. J Clin Endocrinol Metabol. 2013;98(11):E1813–E1819. 10.1210/jc.2013-1653. 10.1210/jc.2013-1653 PMC5399523 24030942

[8] Eng C, Clayton D, Schuffenecker I (1996). The relationship between specific RET proto- oncogene mutations and disease phenotype in multiple endocrine neoplasia type 2. International RET mutation consortium analysis. JAMA.

[9] Cohen MS, Moley JF (2003). Surgical treatment of medullary thyroid carcinoma. J Intern Med.

[10] Ungureanu S, Şipitco N, Alexa Z, Gonţa V, Bujac M, Parnov M, Romanenco R (2020). MEN 2A syndrome - multiple endocrine neoplasia with autosomal dominant transmission. Int J Surg Case Rep.

[11] Lenders JW, Duh QY, Eisenhofer G (2014). Pheochromocytoma and paraganglioma: an endocrine society clinical practice guideline. J Clin Endocrinol Metab.

[12] Huang Y, Wang LA, Xie Q (2018). Germline SDHB and SDHD mutations in pheochromocytoma and paraganglioma patients. Endocr Connect.

[13] Neumann HP, Bausch B, McWhinney SR (2002). Freiburg-Warsaw-Columbus Pheochromocytoma Study Group. Germ-line mutations in nonsyndromic pheochromocytoma. N Engl J Med.

[14] Aguiar RC, Cox G, Pomeroy SL, Dahia PL (2001). Analysis of the SDHD gene, the susceptibility gene for familial paraganglioma syndrome (PGL1), in pheochromocytomas. J Clin Endocrinol Metab.

[15] Boedeker CC, Ridder GJ, Schipper J (2005). Paragangliomas of the head and neck: diagnosis and treatment. Fam Cancer.

[16] Guilmette J, Sadow PM (2019). A guide to Pheochromocytomas and Paragangliomas. Surg Pathol Clin.

[17] Erickson D, Kudva YC, Ebersold MJ (2001). Benign paragangliomas: clinical presentation and treatment outcomes in 236 patients. J Clin Endocrinol Metab.

[18] Neumann HP, Pawlu C, Peczkowska M (2004). European-american Paraganglioma Study Group. Distinct clinical features of paraganglioma syndromes associated with SDHB and SDHD gene mutations. JAMA.

[19] Lenders JW, Duh QY, Eisenhofer G (2014). Pheochromocytoma and paraganglioma: an endocrine society clinical practice guideline. J Clin Endocrinol Metab.

[20] Capra AP, La Rosa MA, Briguori S et al. Coexistence of Genetic Diseases Is a New Clinical Challenge: Three Unrelated Cases of Dual Diagnosis. Genes (Basel). 2023;14(2):484. 10.3390/genes14020484. 10.3390/genes14020484 PMC9957527 36833411

[21] Williams MD (2017). Paragangliomas of the Head and Neck: an overview from diagnosis to Genetics. Head Neck Pathol.

[22] Offergeld C, Brase C, Yaremchuk S (2012). Head and neck paragangliomas: clinical and molecular genetic classification. Clin (Sao Paulo).

[23] Tischler AS (2008). Pheochromocytoma and extra-adrenal paraganglioma: updates. Arch Pathol Lab Med.

[24] Dana Erickson YC, Kudva MJ, Ebersold et al. Benign Paragangliomas: Clinical Presentation and Treatment Outcomes in 236 Patients. J Clin Endocrinol Metabol. 2001;86(11):5210–5216. 10.1210/jcem.86.11.8034. 10.1210/jcem.86.11.8034 11701678

[25] Bandara AB, Drake JC, Brown DA (2021). Complex II subunit SDHD is critical for cell growth and metabolism, which can be partially restored with a synthetic ubiquinone analog. BMC Mol Cell Biol.

[26] Benn DE, Robinson BG, Clifton-Bligh RJ (2015). 15 YEARS OF PARAGANGLIOMA: clinical manifestations of paraganglioma syndromes types 1–5. Endocr Relat Cancer.

[27] Wohllk N, Schweizer H, Erlic Z (2010). Multiple endocrine neoplasia type 2. Best Pract Res Clin Endocrinol Metab.

[28] Loveday C, Josephs K, Chubb D et al. p.Val804Met, the Most Frequent Pathogenic Mutation in RET, Confers a Very Low Lifetime Risk of Medullary Thyroid Cancer. J Clin Endocrinol Metab. 2018; 103(11):4275–4282. 10.1210/jc.2017-02529. 10.1210/jc.2017-02529 PMC6194854 29590403

[29] Gómez K, Varghese J, Jiménez C. Medullary thyroid carcinoma: molecular signaling pathways and emerging therapies. J Thyroid Res. 2011;815–26. 10.4061/2011/815-826. 10.4061/2011/815826 PMC3112527 21687607

[30] Shepet K, Alhefdhi A, Lai N (2013). Hereditary medullary thyroid cancer: age-appropriate thyroidectomy improves disease-free survival. Ann Surg Oncol.

[31] Harari A, Inabnet WB (2011). 3rd malignant pheochromocytoma: a review. Am J Surg.

[32] Pacak K, Eisenhofer G, Ilias I (2009). Diagnosis of pheochromocytoma with special emphasis on MEN2 syndrome. Horm (Athens).

[33] Brunt LM, Lairmore TC, Doherty GM (2002). Adrenalectomy for familial pheochromocytoma in the laparoscopic era. Ann Surg.

[34] Alevizaki M (2013). Management of hyperparathyroidism (PHP) in MEN2 syndromes in Europe. Thyroid Res.

[35] The gnomAD Database. https://gnomad.broadinstitute.org/variant/10-43119548-G-A?dataset=gnomad_r4. Accessed 19 Apr 2024.

[36] The gnomAD Database. https://gnomad.broadinstitute.org/variant/11-112087868-C-T?dataset=gnomad_r4 Accessed 19 Apr 2024.

[37] Moo-Young TA, Traugott AL, Moley JF (2009). Sporadic and familial medullary thyroid carcinoma: state of the art. Surg Clin North Am.

[38] Loveday C, Josephs K, Chubb D, Gunning A, Izatt L, Tischkowitz M (2018). p.Val804Met, the most frequent pathogenic mutation in RET, confers a very low lifetime risk of medullary thyroid Cancer. J Clin Endocrinol Metab.

[39] Rich TA, Feng L, Busaidy N, Cote GJ, Gagel RF, Hu M (2014). Prevalence by age and predictors of medullary thyroid cancer in patients with lower risk germline RET proto-oncogene mutations. Thyroid.

